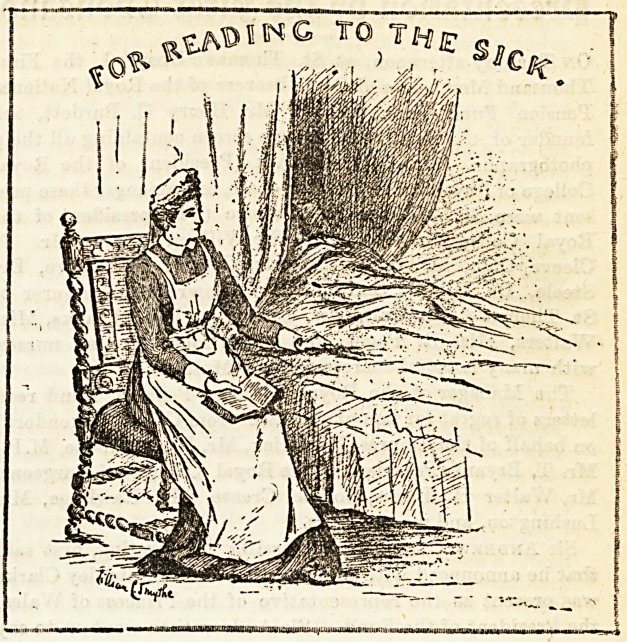# Extra Supplement—The Nursing Mirror

**Published:** 1891-03-28

**Authors:** 


### "The Hospital" Nursing Mirror.

Being the Extra Nursing Supplement of "The Hospital" Newspaper.

Contributions for this Supplement should be addressed to the Editor, The Hospital, 140, Strand, London, W.C., and should have the word "Nursing" plainly written in left-hand top corner of the envelope.

### En Passant.

SHORT ITEMS.—Whitehaven Guardians, by a narrow majority, have decided not to have trained nurses at the infirmary.—The sum of £550 was realised and transferred to the general funds of the Royal Free Hospital last year by its private nursing institution; yet there are only 12 private nurses.—The Staffordshire Institution for Nurses, of which Miss Shirley is Lady Superintendent, issues a good report. There are 69 nurses on the staff, 16 of whom are district nurses, and they earned £3,514 last year.—The Brassey Holiday Home celebrates its anniversary next week, and many nurses are going down to St. Leonard's for the occasion.—The Workhouse Infirmary Nursing Association is sending the first trained nurse to Cockermouth Infirmary. —Dewsbury Infirmary has approved a draft scheme for employing an extra nurse either at the Corporation Hospital or in the district, as she may be needed.

THE JUBILEE INSTITUTE.—The Queen has approved the report of the Jubilee Institute for 1890. The number of nurses on the roll is 94, and there are 15 associations affiliated to the Institute. Badges have been instituted of three kinds—(*a*) The badge to be worn by all Queen's nurses. (*b*) A badge to be given to chief officials, at the discretion of the Council. (*c*) A pendant for distinguished service which the Council may deem deserving of special recognition. The badge (*a*) is given on condition that it shall be returned if the nurse ceases to nurse the sick poor, or works under associations not affiliated to the Institute. The Council have thought it wiser not to press the adoption of the uniform for Queen's nurses. The report goes on to say "The work of inspection is carried out in all places where the institutions or associations receive any aid from the Queen's Institute. This is one of the most important parts of our work, and is very efficiently undertaken by our General Inspector, who, by her great tact and sympathy, as well as by her thorough knowledge of her work, has made the work of inspection to be greatly valued, even where there was a disinclination to submit to it."

RASH ACCUSATIONS.—Hospitals are indeed horrible places if all the stories told about them are true, but truth is frequently absent from every-day conversation: we have long since given up believing what we hear, and the following story shows that we are justified. On March 10th we received a letter from a nursing sister, giving minute details of a disgraceful scene in a hospital ward; as a matter of fact, it was the Middlesex Hospital which was singled out for reprobation, and we were asked to print the letter. We wrote and asked if we might submit the letter to the hospital secretary. We received permission; the letter was sent, the secretary wrote back asking the date of the occurrence, and other particulars. The request was forwarded to the nursing Sister, and her answer was an apology and a request that her first letter might be withdrawn! The whole scandal collapsed when inquired into. Now this is very serious. We cannot help wondering how many people heard and believed that story before it was forwarded to us. Undoubtedly the Sister believed it, but she had never thought of verifying it. We would strongly impress on everyone that the right thing to do when they are told a "hospital scandal" is not to repeat it at afternoon tea, but to go, or write, straight to the secretary of the institution, and find out if there is any truth in the story.

PORTSMOUTH NEWS.—Mr. H. Rundle, F.R.C.S., has lately commenced a series of nursing lectures at the Portsmouth Hospital. There was a good attendance, and Admiral Sir Henry Chad moved a vote of thanks to the lecturer. Dr. Lloyd Owen seconded, and hoped the lecture would be printed and circulated among the nurses. They had in that hospital as good a staff of nurses as they could find in any hospital, and they owed much of this to Miss Tillett, who had given great attention to this part of her work since she had been Matron. The Portsmouth Society for Nursing the Sick Poor has lately issued its report; the nurses attended 203 cases last year, at a cost of 8d. a visit. The prospects of the Society are fairly good, as 78 names have been added to the subscription list.

STARTING A NEW SOCIETY.—A public meeting was held at Longton lately to inaugurate a sick nursing association, the Mayor in the chair. Mr. Dawes moved: "That an association be formed, to be called 'The Longton Sick Nursing Association,' for the employment of two trained nurses, to visit the sick poor of the borough." He knew from experience how difficult it was for medical men to realise their aim on entering the houses of the poorer classes. The mothers were so inexperienced, and there was no one to carry out the instructions given by the doctors. He believed the sum of money required for the support of two trained nurses, who would be able to do an immense amount of good, would only amount to about £200 a-year. Dr. Nicholls seconded the resolution, and said that an erroneous impression had got abroad as to the view the medical men of the town took of the formation of this society. Among his professional brethren there was not one who would not be heartily glad to see this nursing society established as soon as possible. Other speakers followed, all in praise of the scheme, and an influential committee was appointed.

INFECTED CLOTHING BY PARCELS POST.—Serious allegations were made at the Guildford Rural Sanitary Authority on Saturday last, in regard to the transmission of infected clothing by Parcel's Post. The facts of the case, as reported by the Isolation Hospital Committee, were to the following effect: A parcel of clothing was received by the Isolation Hospital to be disinfected. The original sender was Nurse Welch, of Haslemere, who had been attending a scarlet fever case at Churt, near Farnham; but from inquiries which were made it transpired that she erroneously sent the clothes to the Surrey County Hospital at Guildford, instead of to the Isolation Hospital, through the medium of the Parcels Post. The Postal authorities had been communicated with, but they were not disposed to take any action. A letter received from Colonel Lamb, Secretary of the Surrey County Hospital, stated that the parcel arrived at the Hospital (date not remembered) and was opened by the matron, who, finding that it contained infectious clothing, repacked it tightly in thick brown paper and sent it by Parcel's Post to the Isolation Hospital as no disinfecting was done at the County Hospital. Nurse Welch had also been informed to that effect. It did not transpire how the clothing reached the County Hospital. Mr. H. M. Weston, J.P., C.C., moved that under section 126, clause 3 of the Public Health Act, legal proceedings should be instituted against the Matron of the hospital. Although it might be an extremely painful thing to prosecute such a respected person as the Matron of the County Hospital, yet he thought in the interest of the public health they ought not to refrain from exercising their proper authority in the case. Lieutenant-Colonel Tredcroft J.P., C.C., seconded.

### Lectures on Surgical Ward Work and Nursing.

By Alexander Miles, M.B. (Edin.), C.M., F.R.C.S.E.

Lecture XVIII.—After Operation.

While the operation is going on the patient's bed should be prepared for him, fresh sheets, blankets, and pillow-cases, thoroughly aired, being put on it. Next the mattress is put an ordinary sheet, and over this a large, thick macintosh, and then the draw-sheet, on which the patient is laid. For the first few hours after coming from the theatre he should have a blanket next him. This may be removed and replaced by a sheet when the shock has passed off. Outside of this one or two blankets and a cover make up the complement. Before the patient is put into this bed it should be thoroughly heated up by means of several hot bottles placed under the blankets. On the return of the patient to the ward, the blankets and hot bottles should be lifted quite off the bed. As gently and with as little disturbance as possible the patient is to be lifted on to the bed, and the blankets and bottles replaced at once, care being taken that a layer of blanket always intervenes between the bottle and the skin of the patient lest he be burned and, on account of his semi-anæsthetic condition, give no sign. If necessary, a cage is put in over the wounded part, to remove the weight of the bed-clothes from it. Only one pillow should be put under his head at first, and blocks should be ready with which to raise the foot of the bed should he give signs of syncope.

Chloroform Sickness.—As precautions against after sickness and vomiting a towel may be spread over his chest, and a solution tin placed within easy reach of the nurse. On no account may the nurse leave the patient, even for a moment, till he is thoroughly out of the chloroform. In the less serious surgical operations in which the patient does not suffer much from shock the most troublesome immediate after-effect, as a rule, is this chloroform sickness. Patients vary very much in the amount of suffering this causes them, as well as in the benefit they derive from the various methods of treatment. A good general rule is to allow the patient no food or drink for about four hours after regaining consciousness. Should he remain free from sickness during this time he may have small pieces of ice to quench the thirst of which he will most likely complain. This may be followed by iced milk, potash, or Valentine's meat juices. If the sickness persist and be severe you must first try starvation as a means of treating it, and should this fail the following may be tried in succession: (1) sucking small pieces of ice, (2) sipping very hot water, (3) hot fomentations applied over the region of the stomach, and (4) mustard and linseed poultice in same place. The administration of such substances as bismuth, dilute hydrocyanic acid, usually given to allay vomiting, are, as a rule, of no use in this condition. Morphia and cocaine given internally, however, are sometimes useful. They must never be given, of course, except by order of the doctor. In some cases the sickness yields to none of these means, and the vomiting continuing prevents the nutrition of the patient, and may even prove fatal. In such cases every effort must, of course, be made to avert this calamity, and one means of feeding the patient is by *nutrient enemata*. The rectum having been washed out with a small enema of hot water or boracic lotion, a small nutrient enema may be given. It must not exceed two ounces in quantity, or it will not be retained. A good recipe is—Take of brandy, one and a half tablespoonful; Valentine, one dessertspoonful; warm water, one tablespoonful. This may be repeated in two hours, if necessary. It is often necessary to give some more direct cardiac stimulant than the brandy, and I have found that five or six minims of tincture of strophanthus, added to the enema, seems to act very well. Ordinary beef tea is not absorbed by the rectal mucous membrane, and should therefore not be given as a nutrient enema. The thirst in these cases of persistent sickness is often alleviated by about two ounces of warm water thrown into the rectum.

Other Dangers Arising after Operation. — Further precautions must be taken, of course depending on the nature of the operation and the condition of the patient after it. For instance, in cases of severe railway smash where limbs have been amputated, there is considerable danger of *reactionary hæmorrhage*. By "reactionary hæmorrhage" is meant bleeding from a wound within the first few hours after operation, *i.e.*, during the period of "reaction." It may be due to a ligature slipping, or to some paralysed vessel which has not been ligatured regaining its tone after the patient begins to recover from the shock of the operation. In these days of anæsthetics and bloodless surgery this form of hæmorrhage is fortunately less common than it used to be when speed in operating was the great desideratum, but it does occur every now and again, and you cannot be too careful in watching for it. In any case in which it is more than usually likely to occur, it should be the doctor's duty to place a tourniquet in position, ready for the nurse to screw up tight should occasion arise; but if this be not done, then the nurse must rely on her knowledge of anatomy and surgical principles to compress the main artery above. That a hæmorrhage may not be allowed to go on for a time unobserved, the nurse should frequently examine the dressing, especially at its most dependent part, where the blood will first show itself. Red bed jackets or gowns should never be worn by operation cases, as hæmorrhage on to them may remain unobserved. In most cases, however, it is for serous or purulent discharge rather than for blood that a look-out must be kept, and this is scarcely less important. In all cases, then, keep a sharp look-out on the dressing for the first twelve hours, so that no form of discharge may come through without your noticing it; and should either occur, your first duty is to summon the doctor, meanwhile doing whatever is necessary to prevent the mischief going further.

Pulse and Temperature.—The patient's pulse and temperature should be taken as soon as he is out of the anæsthetic, and marked on the chart with a note, "after operation," as these observations form a point of comparison with others to be made subsequently.

Subsequent Dressing After Operation.—How often is it necessary to dress a wound? The less it is touched the better. The aim of antiseptic surgery is towards no dressing at all, that is to say, healing under the original dressing applied at the operation. Unfortunately we have not yet reached the stage when this is possible in all our cases, although it is often attained, and we must now consider the points which are to guide us in deciding as to whether or not we shall dress a particular case.

(1) In the first place we must always dress a wound *if the discharge has come through the dressing*. This is necessary because of the danger of septic mischief reaching the wound by way of the discharge. The nurse should endeavour to anticipate the discharge reaching the surface by frequently examining the most dependent part of the dressing, folding aside a few layers of the bandage. Should she find that it is within a short distance of the surface, a large pad of antiseptic wool, dusted over with iodoform, should be at once placed over the area, till preparations have been made for re-dressing the whole wound. This pad, you must bear in mind, is only a temporary protection, and is not to take the place of a fresh dressing, as the organisms may have already gained access to the discharge and will be "en route" for the wound.

(2) Even when "nothing is through" as the phrase goes, *if the patient complain of much pain* in the part you should dress the wound at once. The discharge may not be getting away, a drainage tube may have become blocked, a stitch may be too tight, and so on, and it is only by redressing that you can either find the cause of the pain, or relieve it.

(3) If on the second or third day after operation the patient's temperature rushes up to 102 deg. or 103 deg. F., and no general condition explains the rise, you will do well to dress, as the probability is the wound has gone septic, and if so the sooner it is dressed the better. Of course, an unexplained rise of temperature at a late date should be investigated in the same way.

(4) If there be *a disagreeable odour* from the dressing it is safer to dress than to leave the wound.

(5) When *drainage tubes* are employed it is necessary to dress the wound after a few days to remove or shorten them, even should everything be going well otherwise.

### The Eastern Hospital Scandal.

The inquiry was proceeded with last week. Some amusement was caused by the following remarks during Mr. Henry Hill's evidence: Cross-examined by Mr. Gye: How did you come to give evidence?—I do not know. I received a subpœna. I never saw Mr. Simpkin in my life. I have not been to the Salvation Army Rescue Home in connection with this inquiry. I do not know who subpœnaed me. Mr. Hedley: It came from me. Mr. Eldridge: My friend will get tired of this "conspiracy" bubble before he is done. Amy Sara, formerly a nurse at the Eastern Hospital, and now of the Salvation Army, stated that she entered the Eastern Hospital as assistant-nurse on the 23rd August, 1887, and left on the 21st June, 1890. The food was very inferior. Poultices were made in the children's bread and milk basins. Little notice was taken of her complaints. Dr. Collie was not in the ward every day. On one occasion, when the day-room of St. George's was first opened, Nurse Halkin said she would not allow the patients to sleep in the day room, as the beds put there were scarlet fever beds, and they had not been disinfected. They slept there the next night. They were convalescent diphtheria patients. Scarlet fever broke out among them. One child died in consequence. Another witness stated that she saw a nurse going all round one of the wards with a sponge or flannel and a towel in her hands, wiping the faces of several children with the same article. Some of the children had sore eyes, sore mouths, and sore faces generally. Annie Mowberry, an assistant-nurse at the Eastern Hospital from December, 1887, gave corroborative evidence as to the bad food.—Cross-examined: I came here out of curiosity. Nobody asked me to come. At first I did not intend to give evidence, as I did not want to be mixed up with it, and I did not want my name to get into the papers. On thinking it over, however, I thought I had been unjustly treated at the hospital, and, knowing the evidence which has been given was true, I thought I would corroborate it. Mrs. Annie Harrington, a former nurse, was next examined, and said she knew Simpkin when he was in the hospital. He complained of everything. Witness did not pass them on, as she did not want to make mischief. They were well founded. She knew it was wrong not to pass on the complaints, but she had no one to complain to. Mr. Gye: Do you say that if you had made a report to the Superintendent Nurse, Dowsett, she would not have passed it on?—No, she would not. Re-examined: When I complained to Nurse Dowsett she flew in a temper and woke the patients up. I reported the matter to the Matron, who sent me to Dr. Collie with a note. I heard no more of it. Nurse Dowsett frequently went into tempers. She was not always capable of controlling her temper, and sometimes smelt of liquor. During the eleven months she was in Mercy Ward she might have seen Dr. Collie seven or eight times. On one or two occasions she thought more might have been done for the patients medically. She referred to children suffering from abscesses, which she thought ought to have been opened. She mentioned this to the day nurse, who replied that it was too much trouble for Dr. Collie, and that he left in a furious temper whenever she spoke to him about the matter. Two days after this a lady doctor took charge of the ward.—The inquiry was adjourned.

### Death in Our Ranks.

On March 17th, at Lyme Regis Cottage Hospital, Mrs. Harriet Neal, aged 66 years, died from blood poisoning. Mrs. Neal was a nurse in the Lyme Regis Hospital for eight years, but latterly she had been working in the town on her own account. She was admitted to the hospital as a patient two months ago, and throughout her long illness was most grateful for all that was done for her.

### THE INVITATION.

Are you sick and wretched, restless alike with pains of body and mind? Then listen to the gracious message, "Come unto me all ye that are weary and heavy-laden, and I will refresh you." Some of us are so foolish that we try to find consolation and refreshment from any source rather than Christ; we take our fill of pleasure, and hew out for ourselves fountains, empty fountains that can hold no water. But Wisdom cries to the starving, craving soul, "Come, eat of my bread, and drink of my cup which I have mingled, forsake the foolish and live; and go in the way of understanding." Let us go with eagerness, whether in health or sickness, and set our faith firmly on Him who alone is able to save, and having tasted that the Lord is gracious, we will grasp the hand He holds out to us, and which we dare not let go.

For this dear Lord will have no half-hearted allegiance, no choosing what we will do, or what we will not do; we must be His obedient, loving children or His enemies. "He that is not for Me is against Me," "Ye cannot serve God and Mammon." These are His own words, while at the same time He presses us to come into His fold.

How dearly He loves us! How freely He shed his life-blood to save us from our sins! Shall we then any longer delay? No, at once and for ever we will accept His invitation. Now our pains are as nothing, for He soothes them; our trials are light, for He bears them; and on the Cross He took the burden of our sins and the punishment of them.

The tremendous sacrifice of His death shall not be useless; the price He paid for our salvation shall never be thrown away by our own ingratitude or carelessness.

"Weary of earth and laden with my sin,
I look at Heaven and long to enter in;
But there no evil thing may find a home,
And yet I hear a voice that bids me 'come.'

"It is the voice of Jesus that I hear,
His are the Hands stretched out to draw me near,
And His the Blood that can for all atone,
And set me faultless there before the Throne."

### Appointment.

Holherton Institution, Antigua.—Miss Kate Freeth is going out to the Leeward Isles to take charge of this institution. Miss Freeth trained at the London, worked for the Kent Nursing Institution, and has lately superintended a private medical and surgical home. Her testimonials are excellent, and we heartily wish her success and happiness in her new position.

### Presentation by the First Thousand.

On Tuesday afternoon, at St. Thomas's Hospital, the First Thousand Members and Purse-bearers of the Royal National Pension Fund presented to Mr. Henry C. Burdett, the founder of the Fund, a handsome screen containing all their photographs. Sir Andrew Clark (President of the Royal College of Physicians) took the chair, and amongst those present were Sir William MacCormac (Vice-president of the Royal College of Surgeons), Sir William Moore, Mr. F. Cleeve, C.B., Mr. G. H. Makins, Colonel Montefiore, Dr. Steele, Dr. Gilbart Smith, Mr. Wainwright (Treasurer of St. Thomas's Hospital), Mrs. Suckling, Mrs. Franks, Miss Walters, Miss R. Paget, and about four hundred nurses, with many hospital Secretaries and Matrons.

The Manager of the Royal National Pension Fund read letters of regret for non-attendance from Count Seckendorff, on behalf of the Empress Frederick, Mr. W. Rathbone, M.P., Mr. T. Bryant, President of the Royal College of Surgeons, Mr. Walter H. Burns, Colonel Crease, Mr. Rawlings, Mr. Lushington, and many nurses.

Sir Andrew Clark, in addressing the meeting, first said that he announced with pleasure that Colonel Stanley Clarke was present as the representative of the Princess of Wales, the President of the Fund. Sir Andrew then went on to say that the presentation about to be made was a happy acknowledgment of the fact that about the largest and best thing which any man, or body of men, could have devised for the benefit of nurses had reached a successful climax. We know what an indefatigable worker Mr. Burdett is, and that what he takes up he always carries through; he thought of doing something for nurses, and the best he could do, as I said before, was to found this Fund. But it was a very difficult thing to do; it wanted money; and Mr. Burdett had to raise somehow a sufficient sum of money to put it on a secure footing, before asking nurses to come forward and subscribe to it. He raised £50,000, and how he did it I do not know; but he did, and, practically, without noise or fuss or public appeal of any sort. In spite of various antagonisms and misrepresentations, 1,700 nurses have thrown in their lot with the Fund; they have also added or collected subscriptions till the invested capital is now £90,000. This is simply marvellous, and I do not know that such another record exists. It was just like women to think that when this Fund was established they should make some acknowledgment of Mr. Burdett's labours. Three of the nurses formed themselves into a committee—Miss Belcher, Miss Evans, and Miss Hale—and I will leave one of their number to describe this screen.

Miss Mary M. Belcher, on behalf of the Screen Committee, said it was not the thought of three, but of dozens of nurses that they should like to give Mr. Burdett their photographs usefully framed. The three members of the committee simply received the subscriptions, which were limited to one shilling each. The Princess of Wales very kindly sent us her autograph portrait, and everybody seemed most delighted to do what they could to express their gratitude to Mr. Burdett.

Dr. Bristowe, Chief Physician of St. Thomas's Hospital, said that he had long been connected with the Fund, and believed that what had been done would be of the greatest benefit to nurses.

Mrs. Dacre Craven, as the oldest Nightingale and trained nurse in England, said she was proud to have been chosen to present the screen. The sheet covering the screen was then withdrawn, and displayed a very handsome mahogany screen in five partitions, and completely covered by the photographs of the First Thousand members of the Fund. In the middle of the screen were large panel portraits of the Prince and Princess of Wales and of the late Mr. Junius S. Morgan. The workmanship of the screen is of rare excellence, and confers the greatest honour upon the maker, Mr. Roskilly, 7, Berkley Road, Regent's Park Road, N.W.

Mr. Burdett said: I have to thank you most heartily for the spontaneous kindness you have shown me to-day, and for the beautiful screen which you have presented to me. That screen will be very highly prized, not only by myself, but by my children, who will, I know, regard it as a precious heirloom so long as our family lasts.

Every man in this life is charged with responsibility to a greater or a less extent. When God, in His providence, reveals to a man that a particular work is good and that it is necessary to the well-being of those amongst whom his lot has been cast, he who puts such thoughts away from him, and declines to spend and be spent in the cause, incurs a responsibility which no true man ever consents to face.

From 1868 to 1880 I worked in the hospitals of this country continuously, side by side with doctors and nurses. They were my colleagues and friends, and in the contest which we waged together against disease and suffering I learned to realise the self-sacrifice attaching to such labours and to know how often nurses and other hospital officials suffer undeserved privations from devotion to duty. However much they might seek to protect others, there was nothing to guard them against disaster in the day of sickness and old age. To realise this anomaly was to wish to remove it. I had been twice struck down by serious illness, contracted in connection with my hospital work and twice, under providence, restored to health, thanks to the devoted attention of my colleagues.

When I was called to other fields of work I felt impelled to labour steadily and continuously until a pension fund had been firmly established which should be so organised as to be capable of providing adequately for the needs of all workers amongst the sick. To-day sees the fulfilment of that work, for the Royal National Pension Fund for Nurses is wide enough and strong enough to be a safeguard and protection for all nurses and hospital officials throughout the British Empire.

Although I may be regarded as the architect of the building, and, perhaps, as captain of the ship, still success has only been possible because the cause has won the support and sympathy of all classes and of every person who has had anything to do with it from first to last. Our gracious Princess and President, the Patron, Patronesses, Vice-presidents, Members of Council, and Officers have all done their utmost to secure the accomplishment of the business in hand, and it has never been regarded as anything but an honour on the part of anyone of them to exercise self-denial, provided only that the Pension Fund might be thereby benefited and helped. Last, but not least, H. M. the Queen has been graciously pleased to command that the Fund shall be called Royal.

A few of my colleagues in this matter stand out prominently. First, there was Mr. Junius S. Morgan, who at the outset, when £20,000 was required, stepped manfully into the breach, and to him and to Lady Rothschild (who cordially endorsed and supported Mr. Morgan throughout) is due the credit of having made the Fund possible. When it was known that Mr. Morgan and Lord Rothschild had each given £5,000, Mr. Hambro (an excellent friend of the Fund) and Mr. Gibbs at once gave the remaining £10,000, so that the whole £20,000 was subscribed within ten days.

As the nurses know already, Mr. Morgan led me to understand that he, himself, would give the whole £20,000, should it be necessary. Their grateful hearts showed how they appreciated his action and kindness when they raised, within the short period of six weeks, upwards of £2,200 to form the nucleus of a memorial to his name. This spontaneous action on their part so touched the hearts of Mr. Morgan's family that my devoted colleague and friend, Mr.

Walter H. Burns, the Chairman of the Fund, has now been the means of raising the Benevolent Fund to £10,000, so that no nurse who joins need have any fear should she be temporarily prevented from paying her premiums through unforeseen circumstances, because the income of the Benevolent Fund, say £400 a-year, will be there to shield her from temporary embarrassment. Dr. Bristowe, too, than whom there is not a more staunch or truer man in the whole profession of medicine, has done yeoman's service in conjunction with Mr. Thomas Bryant, as Honorary Medical Officers to the Society.

Such, then, is a brief history of the Fund. It shows that to others, rather than to myself, must the credit, in a large measure, belong. Nevertheless, so far as I have been able to help the Fund I am thankful, and I thank you for this splendid testimony of your appreciation and good-will. May I in return stretch out to you the right hand of fellowship, and assure you, and through you, all nurses wherever they may be placed, and all hospital workers too, that I regard it as my privilege and pleasure to aid them at all times to the best of my ability. I, therefore, hope you will not fail to grasp the hand freely, as it is freely offered you.

This beautiful screen will, as I have before said, be treasured in my family as an heirloom. When placed in my library it will protect me from draughts, and by inspiring me with confidence will cause the shafts of one's adversaries to fall harmless and unnoticed. So I hope the Pension Fund may protect you, and all nurses and hospital workers of every grade, from the hardships which must otherwise come almost as a matter of course to each of you who faithfully endure to the end.

Once more I thank you from the bottom of my heart.

Dr. Steele, Sir William Moore, and others spoke, and then the nurses were invited on the platform to inspect the screen. We are requested to state that for the next fortnight the screen will be on view to any nurse applying at The Lodge, Porchester Square, W., between the hours of 10 a.m. and 6 p.m. The Royal Oak and Queen's Road Stations are within three minutes' walk of this house. The Princess of Wales has, we understand, intimated her intention of receiving the Second Thousand when the Fund can boast that increase of members.

### Notes from Australia.

(*By Our Own Correspondent.*)

Melbourne, February 7th.

Mrs. Strong and Miss Martelli have at last won their point, and on January 21st their private hospital was opened with ceremony. The Board of Public Health refuse to allow them to take typhoid cases, which is a great pity, for it is very prevalent again, and of course good nursing and quiet are everything in typhoid.

A presentation of a silver salver, suitably inscribed, was lately made to Mrs. H. Tredgold Strong, late Matron of the Alfred Hospital, at Rokeby, St. Kilda, now occupied by Mrs. Strong and Miss Martelli as a private hospital. The gift had been subscribed for by twenty of the pupil nurses trained during the stay of Mrs. Strong at the Alfred Hospital. The salver bore the names of the twenty subscribers, and was presented by three of their number, Misses O'Sullivan, Bolton, and Elms.

A series of papers by C. Candler on "Koch's Proposed Cure for Consumption" have been appearing in the *Argus*, and have excited much interest. Mr. Candler is one of the City coroners, but he lives a very quiet life at the Melbourne Club, and is devoted to scientific pursuits. Mr. Candler says sunlight is the proper cure for consumption: "It is a sequence that if all human beings slept in well-sunned rooms consumption would cease and determine. There would be an end of the disease. And if it is a fact that consumptives may be cured by sun-treatment, it is an inference from the fact itself, no matter what the explanation of the fact may be, that by the universal adoption of the plan of sun-treatment consumption would be entirely suppressed. In fine, it is as certain that sun-prevention is included within the principle on which the sun-cure is based as that the greater includes the less. It is, moreover, evident that whether the principle underlying sun-treatment has or has not been made out, the treatment, if effectual in the cure, will be effectual in the prevention of consumption." I remember in Miss Nightingale's "Notes on Nursing" she says that more people die on the north side of a street in England than on the south, and gives as a reason sunshine or its absence. On January 6th, at the ordinary meeting of the Committee of Melbourne Hospital, a letter was read from Mr. E. Arundel Carttar, who is coroner for the county of Middlesex, England, referring to the death of his sister, a nurse in the institution, in August last from diphtheria, and stating that, from the reports he had received from independent persons, he was obliged to come to the conclusion that the deceased had been neglected whilst ill. The Chairman said the matter had been fully inquired into at the time. Dr. Moloney, who attended the case, said that there had been no neglect, and that he was quite satisfied that the nurse had received every care. Dr. Molloy, the resident surgeon, informed the Committee that he had visited the patient half-a-dozen times a day, and Dr. Moloney, in whose care she was had visited her three or four times daily. It was resolved to send the reports received from the doctors to the writer, and also the report of the autopsy. Miss Carttar had complained shortly before her death of want of food, and when a friend went to inquire after her, he was unable to see the secretary or gain information for half an hour: then he was informed of Miss Carttar's death, and that the funeral was just about to take place.

The Lady Superintendent of the Melbourne Hospital drew, the attention of the Committee to the accomodation provided for those nurses who are engaged nursing contagious cases. During the past week there had been seven nurses working amongst diphtheria and scarlet fever. It was only possible for four of these to be in a measure separated from the others. The three others were obliged to be mixed up with the general nursing staff, both at meals and in their bedrooms. There was some idea of using the new refractory wards for isolated cases, and, if so, she was afraid it would be impossible for her to supply the nurses who undoubtedly would be required for those cases, and the whole question of the accommodation of the nursing staff should be carefully and at once considered. The nurses at the Melbourne are very irate just now at the system of espionage maintained by the secretary who visits the wards at uncertain times. Mr. Williams is enthusiastic, and in many ways an excellent worker; *it is a pity* he has so little tact, and fails to see that the nurses should be responsible to the Lady Superintendent alone.

Dr. Goldstein on the question of hospital abuse proposes that inquiries as to the circumstances of out patients should be made at irregular intervals by the C.O.S. The fear of detection would keep away those few who now avail themselves of charity they do not need.

The Medical Society of Victoria has held its annual meeting, and elected Dr. Hinchcliff President for the current year. In his retiring address Dr. Jackson dealt with the Charities Commission, private hospitals, Koch's cure, and the proposed union of our two medical societies.

The Victorian Branch of the British Medical Society has held a dinner, Dr. Le Fevre in the chair, and about one hundred members were present. Mr. Staughton, M.L.A., proposed "The Medical Society of Victoria." He thought that if the two medical societies in this colony were united, they would work better than they could separately.

Some startling facts are just issued in the record of the Melbourne morgue for 1890. Inquests were held on 416 bodies, and in 38 cases a verdict of suicide was returned. There were 13 murders and 153 fatal accidents; alcoholism claimed 51 victims by sudden death. And these drink statistics are interesting: Of the eight suicides by shooting four were directly traceable to drink, all the six self-poisoners were drunkards, and all those who drowned themselves had vainly tried to drown their sorrows while they lived. Of the eight who hanged themselves three were drunkards; of the eight who cut their throats four were of the same inclination; one of those who lay down in front of a railway engine drank heavily; three of the six manslaughtered drank; seven of the 20 accidentally killed by the railways; six of the 15 accidentally drowned; four of the nine tramway victims; 10 of the 23 run over on the streets; 17 of the 28 "found dead"; and on through to the end of the chapter, drink was the ruling passion, the fatal tendency.

### Off Duty

### Through the Hoop.

The wide arena of a circus, dazzling lights and loud braying music, a shifting sea of faces, tier above tier, then a deafening clapping of hands as the Little Wonder entered. A fair-haired mite dressed in a fragment of white mist, her winsome face framed in masses of yellow hair. Dot was the idol of the house. Who among the great crowd would listen as they gazed rapturously upon the gay little sprite, to a pitiful story of severe training, of bitter, jibing words, of hard, brutal blows? Not one, for Dot's radiant face was a flat contradiction. The public, when it chooses, is a big child, and believes only just as much as is its pleasure to believe. Dot was the envy of every other child in the building, and an uproarious greeting was accorded her. With pretty smiles and gestures, the little one showed her gratitude, but only her own tiny ears heard the ring-master's harsh admonition. Perhaps never had Dot gone through her work more gracefully and airily; even the grim tyrant's face was relaxing, when a careless laugh from some one of the audience rang out on the heated air, causing the startled Dot to glance aside, and she missed the hoop, falling, with a dull thud, on the sawdust. For a second, as one man, the huge assemblage held its breath. Then a confused hoarse clamour broke forth; people sprang from their seats, questions were wildly shouted, and directions as wildly offered; a couple of medical students pushed their way through the surging crowd that encircled the silent heap of spangle, white gauze, and golden curls.

"She's not dead, thank God!" breathed out one of the lads sobered into a Solomon.

"Get a mattress!" ordered the other. "The poor little soul must go to St. Jude's straight," and not a precious minute was lost. Before each individual making up the vast assemblage had reached his or her roof-tree, Dot was lying still and grey-white, but with her tender bones all set, in a certain cot that had been yawning for a small occupant.

A long room, very quiet and peaceful, soft-voiced nurses flitting noiselessly from this bed to that, the sunshine making free, to its heart's content, with all objects, human and otherwise, a posy of sweet purple violets lying on the counterpane close to her lips; such was the scene upon which Dot opened her eyes next morning, a strangely different one one from that of the evening before. It was too much exertion to wonder what it all meant, so the child dreamily drank it in without stirring. The rest, the undisturbed peacefulness, was unspeakably beautiful. By-and-bye, when a cool, soft hand was laid upon her brow, Dot spoke.

"I'm glad I've died," she murmured composedly.

"It's *much* nicer than livin'. Mother died long, long ago; perhaps she's in one of those beds over there. She used to tell me about heaven. I know all about it; this is it—a place to lie still and rest. When I was little I thought heaven was through the hoop. . . . He beats me every time I miss the hoop"—there was a faint shudder—"Who's that laughing? Ah-h!"

But as the hours slipped by the excited brain settled down, the scattered little senses gathered themselves together, and Dot understood that she was in the hospital ward, her injuries mending rapidly.

"Do you mean to say the kid will be no use to nobody no more?" a rough voice asked.

"Not to such as you, my man. She has escaped with her life, but she'll be lame all through it." Then the rough voice muttering language that was unorthodox, died away for ever out of Dot's existence, leaving her as a legacy to St. Jude's.

"I think *I* can make room for the sweet little creature," said somebody who had come, day after day, to sit by Dot's bedside, fascinated by the dainty, woebegone face. Somebody was a quaint little maiden-lady—an "alone-standing lady"—thin and spare as a chip, with eyes that squinted, and a pasty face that had never, in its freshest youth, been fair. That is how Miss Mummery looked to earthly eyes. Perhaps to the clearer, purer vision of the angels she was passing beautiful, for they regard the soul within, instead of the outer covering of flesh. Wealth gave the little lady power, and her warm heart moulded that power aright.

"I only do what I can," said she, abashed at praise; "but I'll indulge myself for once; I'll take that human flower home, she'll be a better fad than Puss or Pincher." So it comes to pass that Dot, from a safe haven, looks out, through the hoop of her home in Miss Mummery's heart, upon the outside world.

### Princess Christian's Daughter.

Miss E. Durham, Farringford, Freshwater, Isle of Wight, acknowledges the following additional subscriptions towards a wedding present for Princess Louise of Schleswig-Holstein to be given as a proof of the gratitude of nurses for the interest Princess Christian has ever taken in their progress. Subscriptions will be received until the end of March, and will be acknowledges in these pages. Between March 16th and 23rd the following sums were received:—

Superintendent Rebe, 5s.; Sister M. A. Monk, 2s. 6d.; Anna Jenkins, 2s. 6d.; Nurse Goldfinch, 2s.; Nurses (1s. each) I. F. Parsons, Annie Belcher, Anne Smith (B.N.A.), Sarah Rees, E. M. Dickson (B.N.A.), E. E. Jennings, L. J. Mason, Sarah A. Mallinson, Edith Gates, H. E. M., Emma M. Daws (B.N.A.), A. E. B., Zoe, I. J. Baskin, Mary, Marie Timpe, Afflick, Fenton (B.N.A.), Sayers, and E.

[We would ask any of our readers who intend to subscribe to do so at once, as subscriptions can only be acknowledged twice more in these pages.—Editor.]

### Amusements and Relaxation.

### SPECIAL NOTICE TO CORRESPONDENTS.

First quarterly word competition commenced January 3rd, 1891; ends March 28th, 1891.

Competitors can enter for *all* quarterly competitions, but no competitor can take more than *one first prize* or *two* prizes of any kind during the year.

Proper names, abbreviations, foreign words, words of less than four letters, and repetitions are barred; plurals, and past and present participles of verbs, are allowed. Nuttall's *Standard* dictionary only to be used.

N.B.—Word dissections must be sent in WEEKLY not later than the first post on Thursday to the *Prize Editor*, 140, Strand, W.C., arranged alphabetically, with correct total affixed.

The word for dissection for this, the THIRTEENTH week of the quarter, being "HOLIDAYS."

| Names. | March 20th. | Totals. | Names. | March 20th. | Totals. |
|---|---|---|---|---|---|
| Reynard | — | 77 | Woodbine | — | 25 |
| Reldas | 42 | 576 | Madame B. | — | 25 |
| Tinie | — | 30 | Shakespeare | — | 59 |
| Patience | — | 76 | Smyrna | — | 259 |
| Jenny Wren | 29 | 506 | Southwood | — | 102 |
| Agamemnon | 40 | 567 | Gipsy Queen | — | 21 |
| Wyameris | — | 391 | Snowball | — | 19 |
| E. C. | 37 | 561 | Rita | — | 88 |
| Ecila | — | 283 | Mortal | — | 16 |
| Hope | 40 | 571 | Nurse Annie | — | 15 |
| M. W. | 37 | 557 | Carmen | — | 11 |
| Qu'appelle | 37 | 553 | Grannie | — | 45 |
| Nil Desperandum | 41 | 565 | Amie | — | 30 |
| Lady Betty | 35 | 522 | M. R. | — | 25 |
| H. A. S. | — | | Primrose | — | 24 |
| Sister Jack | — | 62 | Nurse J. S. | 32 | 324 |
| Crystal | — | 203 | B. A. C. | ... | 223 |
| | | | Theta | ... | 118 |

#### Notice to Correspondents.

Second Quarterly Word Competition commences April 4th; ends June 27th, 1891.

## Figures and Tables

**Figure f1:**